# Visual Defects in a Mouse Model of Fetal Alcohol Spectrum Disorder

**DOI:** 10.3389/fped.2014.00107

**Published:** 2014-10-09

**Authors:** Crystal L. Lantz, Nisha S. Pulimood, Wandilson S. Rodrigues-Junior, Ching-Kang Chen, Alex C. Manhaes, Valery A. Kalatsky, Alexandre Esteves Medina

**Affiliations:** ^1^Department of Anatomy, Virginia Commonwealth University, Richmond, VA, USA; ^2^Department of Biology, University of Maryland, College Park, MD, USA; ^3^Department of Pediatrics, University of Maryland School of Medicine, Baltimore, MD, USA; ^4^Universidade Federal Fluminense, Niteroi, Brazil; ^5^Baylor College of Medicine, Houston, TX, USA; ^6^Universidade do Estado do Rio de Janeiro, Rio de Janeiro, Brazil; ^7^Enthought, Inc., Austin, TX, USA

**Keywords:** retinotopy, fetal alcohol spectrum disorders, fetal alcohol syndrome, visual evoked potentials, optical imaging of intrinsic signals, electroretinography, vision, visual cortex

## Abstract

Alcohol consumption during pregnancy can lead to a multitude of neurological problems in offspring, varying from subtle behavioral changes to severe mental retardation. These alterations are collectively referred to as Fetal Alcohol Spectrum Disorders (FASD). Early alcohol exposure can strongly affect the visual system and children with FASD can exhibit an amblyopia-like pattern of visual acuity deficits even in the absence of optical and oculomotor disruption. Here, we test whether early alcohol exposure can lead to a disruption in visual acuity, using a model of FASD to mimic alcohol consumption in the last months of human gestation. To accomplish this, mice were exposed to ethanol (5 g/kg i.p.) or saline on postnatal days (P) 5, 7, and 9. Two to three weeks later we recorded visually evoked potentials to assess spatial frequency detection and contrast sensitivity, conducted electroretinography (ERG) to further assess visual function and imaged retinotopy using optical imaging of intrinsic signals. We observed that animals exposed to ethanol displayed spatial frequency acuity curves similar to controls. However, ethanol-treated animals showed a significant deficit in contrast sensitivity. Moreover, ERGs revealed a market decrease in both a- and b-waves amplitudes, and optical imaging suggest that both elevation and azimuth maps in ethanol-treated animals have a 10–20° greater map tilt compared to saline-treated controls. Overall, our findings suggest that binge alcohol drinking restricted to the last months of gestation in humans can lead to marked deficits in visual function.

## Introduction

Fetal Alcohol Spectrum Disorder (FASD) is an umbrella term for a variety of conditions affecting the children of women who drink alcohol during pregnancy and is currently one of the leading causes of mental retardation in the world. The effects of early alcohol exposure are wide ranging and can vary from subtle behavioral changes to severe cognitive deficits. Sensory processing deficits may exacerbate the neurobehavioral problems observed in FASD, as these subjects often exhibit delays in auditory processing as well as reduced vision acuity (1, 2).

A subgroup of FASD is Fetal Alcohol Syndrome (FAS), which is characterized by the triad of growth deficiency, central nervous system (CNS) problems, and a specific pattern of facial dysmorphology (3–5). The altered facial features, which include small (or lack of) philtrum, short nose, flat midface, and low nasal bridge are caused by a specific effect of alcohol during the gastrulation phase of development (6–8).

The visual system can be particularly affected by developmental alcohol exposure. Children with FASD often present deficits in spatial frequency acuity and contrast sensitivity, strabismus, amblyopia, poor detection of geometric designs, and abnormal saccadic movements (1, 9–13). The type of visual deficit observed is strongly related to the time of alcohol exposure. Similar to the typical FAS facial dysmorphology, gross ocular defects such as microphthalmia and hypoplasia of the optic nerve are caused by alcohol exposure during the first trimester of the human gestation (6, 8, 14).

Hug and colleagues evaluated the visual abilities of a group of children with FAS (15). All the subjects displayed facial dysmorphology and 10 out of 11 had hypoplasia of the optic nerve. They found that kids with FAS showed a reduction in spatial frequency acuity measured by visually evoked potentials (VEPs) and reduced a- and b-waves after electroretinography (ERG) (15). Studies using animal models have been instrumental in investigating the effects of alcohol consumption at different times during pregnancy, corresponding to different periods of fetal brain development (7). For instance, Katz and Fox demonstrated, in an animal model, findings similar to the human study mentioned above (16). They evaluated ERGs in two paradigms of developmental alcohol exposure. In one experimental group, alcohol exposure (liquid diet, 35% alcohol) lasted from the first day of the pregnancy to parturition (roughly equivalent to the first, and most of the second trimester of human gestation). In another experimental group, alcohol exposure began at the first day of pregnancy but was extended to postnatal day 10 (mimicking exposure all through human gestation). Both groups showed a decrease in the amplitude of a- and b-waves in ERGs. These deficits were more evident in low-light conditions (scotopic vision) (16).

While major ocular malformations have clear effects in visual perception, it is not uncommon for children with FASD to show reduced visual function even when these malformations are not evident. In fact, Vernescu and colleagues were able to detect deficits in visual acuity and contrast sensitivity in children with FASD even in the absence of refractive errors (1).

The last months of gestation in humans and the first two postnatal weeks in rodents are crucial for visual system development. During this period, the retina displays “waves” of activity, which are important for the establishment of the topography of the retino-geniculo-cortical pathway (17–20). Also during this period, the retina and most of the CNS are extremely sensitive to alcohol-triggered neuroapoptosis (21, 22). Therefore, we hypothesize that alcohol exposure even restricted to just this period of development will affect visual function. Our prediction is that alcohol exposure in mice during the first two postnatal weeks will disrupt their spatial frequency acuity and contrast sensitivity measured by VEPs; reduce amplitude of a- and b-waves of ERGs and alter cortical retinotopic maps assessed by optical imaging of intrinsic signals.

## Materials and Methods

All procedures described in this paper were approved by the Institutional Animal Care and Use Committee.

### Alcohol exposure paradigm

Visibly pregnant C57/BL6 mice were obtained from a commercial supplier (Harlan), and singularly housed in the university animal housing. Pregnant dams were checked daily until pups were born. Day of birth was designated as postnatal day (P) 0.

We used the same paradigm of alcohol exposure used in a recent study by our lab showing that developmental alcohol exposure leads to impaired visual cortex plasticity in mice (23). Pups received a single injection of 5 g/kg of alcohol (25% ethanol in normal saline i.p.) or an equivalent volume of saline as a control on days P5, 7, and 9. Typically, within a litter (males and females) 2/3 of animals were injected with alcohol and the remaining with saline. Animals were then alcohol-free for the remainder of the study. According to our previous studies, this exposure paradigm leads to blood alcohol levels of 411 mg/dl (1) at 1 h post injection.

### Visually evoked potentials

#### Surgery

Visually evoked potentials are assessed in awake mice through the use of chronic implanted electrodes. For electrode implantation, P21–22 mice were anesthetized with ketamine (120 mg/kg (Bioniche Pharma, Lake Forest, IL, USA) and xylazine 9 mg/kg (Akorn, Inc., Decatur, IL, USA). Once anesthetized, 2% lidocaine jelly (Akorn, Inc., Decatur, IL, USA) was applied locally on the scalp at the incision site. Silver ground electrodes were implanted 1.0 mm caudal from bregma, and 2.0 mm lateral from the midline.

Tungsten microelectrodes (FHC, impedance 0.3–0.5 MΩ) were implanted 3.00 mm lateral of the midline and 0.5 mm of lambda, at a depth of 0.43 mm. Electrodes were secured to the skull with cyanoacrylate glue (Elmers, Westerville, OH, USA). After surgery, the animal was monitored until recovery of righting reflexes and was then given 0.05 mg/kg of buprenorphine (Stokes Pharmacy, Mt. Laurel, NJ, USA) for post-surgical analgesia.

#### Recording

After the implantation of the electrodes, animals were allowed to recover for 48–72 h. After the recovery period, awake animals were habituated on the experimental setup for 45 min 1 day prior to the experiment. VEPs were recorded using XCell-3 amplifiers (FHC, Inc., Bowdoin ME, USA; one for each recording electrode), a 1401 digitizer (CED, Cambridge, England), and Spike 2 software (Cambridge Electronics Design, Cambridge, England). Visual stimulations were presented to each eye individually using a monitor placed 18 cm from the nose of the animal (mean luminance 27 cd/m^2^, area of 15 cm × 31 cm) and controlled by a custom program using MATLAB (MathWorks, Natick, MA, USA) with Psychtoolbox extensions.

### Visual stimuli and analysis

Stimuli consisted of full-field ordinal sine-wave 2 Hz reversing gratings, at 0.05 cycles per degree (cpd) with 100% contrast. VEP measurements were based on the average amplitude of 100 stimulation presentations. Recorded VEP amplitudes were then used to calculate a contralateral bias index (CBI, ratio of contralateral/ipsilateral response amplitude of each animal). CBI results are reported as average CBI and the standard error of the mean (SEM). For spatial frequency acuity measurements, the stimuli consisted of six randomized full-field reversing sine-wave gratings of 0.5–0.02 cpd and an equal luminance gray screen. Spatial frequency acuity measures were based on average VEP amplitude of 100 trials. For contrast sensitivity measurements, six randomized full-field reversing sine-wave gratings, of 0.05 cpd, with equal luminance and contrasts from 100 to 0% were presented. Contrast sensitivity acuity measures were based on average VEP amplitude of 100 trials. All acuity results are reported as the average VEP amplitude in millivolts and the SEM.

### Electroretinography

Mice were dark adapted overnight and prepared for recording the next day under infrared illumination. Animals were anesthetized with a mixture of ketamine/xylazine (150/10 mg/kg; IP), and the pupils were dilated in the dark for a minimum of 10 min with topical eye drops of 1% tropicamide and 2.5% phenylephrine (Bausch & Lomb, Tampa, FL, USA). The head was held steady in a custom nose ring. A drop of 0.9% saline was frequently applied on the cornea to prevent dehydration, also allowing electrical contact with the recording electrode (a gold wire loop). A sterile reference needle electrode (Rhythmlink, Columbia, SC, USA) was inserted under the caudal most portion of scalp, behind the VEP head-stage. Amplification (at 1–500 Hz bandpass, without notch filtering), stimuli presentation, and data acquisition were programed and performed using the UTAS-E 3000 system (LKC Technologies, Gaithersburg, MD, USA) as previously described (24). Scotopic ERG responses were recorded during single 10 microseconds flash presentations at intensity of 1.37 log cds/m^2^. Six responses were obtained at 20-s intervals, and were averaged for each eye. All results are reported as the average response amplitudes in millivolts and SEM.

### Optical imaging of intrinsic signals

#### Surgery

Mice between 25 and 50 days of age were anesthetized with 10% urethane in saline (1.0 g/kg) injected intraperitoneally. A supplementary sedative, chlorprothixene (0.2 mg/mouse i.p.) was administered prior to urethane. Atropine (5 mg/kg) was injected subcutaneously to reduce bronchial secretion and to counteract the parasympathomimetic effect of the anesthetic agents. An incision was made in the scalp, exposing the occipital region of the animal’s skull. A metal plate with a square window in the center was then glued to the skull, positioning the square window above the visual cortex. Agarose (2.5% in saline) was used to fill the square window and topped with a glass coverslip. A craniotomy is not required with this technique because the mouse’s skull is sufficiently transparent for clear imaging.

#### Image processing

The acquired images were used to create the retinotopic maps; the procedures were similar to those described in Kalatsky and Stryker (25). In short, the time series of light reflectance from each pixel was analyzed independently, after filtering (high-pass, boxcar filter, and size two cycles of the stimulus) the fundamental Fourier component was extracted at the frequency of stimulation. These phases and amplitudes were computed from the cosine and sine components and were used to create the maps. The phase maps are the maps of relative retinotopy. To remove the constant bias, a small region away from the visual evoked activity and free of the vascular and other artifacts was selected, and the mean value of cosine and sine components was computed, this two-dimensional vector was subtracted from all pixels of the map. The maps produced by the oppositely moving stimuli (up–down and right–left) were combined, the phase of each pair of corresponding pixels were subtracted and the amplitude average, to yield the maps of absolute retinotopy (elevation and azimuth) see Figure 4A. Finally, to yield maps of the visual angle the phase maps were divided by a constant factor (7.2).

#### Analysis of retinotopic maps

Although the measure used in this study (map tilt, phase scatter, and magnification factor) do not require the absolute value maps we favored this approach because the combined maps have higher signal-to-noise ratio and for easy of comparison to other studies (26).

##### Map tilt

The map tilt was computed as the angle of the representation of the central line of the stimulus monitor on the cortex. The central line (horizontal for the up–down stimulus and vertical for the right–left stimulus) has zero-phase in the absolute retinotopic maps, which is coded in color blue (Figure 4). The angle of the zero-phase line on the cortical surface was computed relative to the mediolateral axis for the elevation maps and the anteroposterior axis for the azimuth maps. Results are reported as the mean angle and SEM.

##### Phase scatter

To evaluate the quality of the maps we used the phase scatter, which was computed as the difference between the phase of a pixel and the mean phase of its neighborhood (including the pixel itself). The neighborhood was defined as the pixels within a circle of radius 2.9 drawn around the central pixels, which resulted in 5 × 5 square footprints. A region with visually evoked activity and free of artifacts was selected. The pixels within the region were ranked by amplitude of response and at most 20,000 pixels within the highest response were selected. The standard deviation of the phase scatter of these pixels was used as the measure of the map quality.

##### Magnification factor

The magnification factor (the amount of visual angle representation per millimeter of cortical distance) was computed along the lines perpendicular to those used for the map tilt calculation (the zero-phase lines), these lines correspond to the steepest progression of the phase change. Two points were selected on the steepest phase ascent line corresponding to approximately ±5° of the visual angle; the cortical distance between these two points was measure (in millimeters). The ratio of the visual angle difference (typically 10 degrees) over this distance yields the magnification factor. Results are reported as the average visual degree per millimeter and the SEM.

## Results

### VEP amplitudes

**Figure 1:**
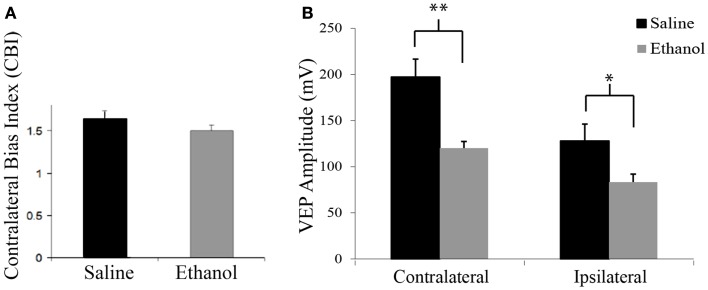
**Contralateral bias index and amplitudes of visually evoked potentials**. **(A)** Alcohol treatment does not affect the eye dominance in the binocular zone of the visual cortex. Note similarity of CBIs between groups. **(B)** Alcohol treatment affects amplitude of visually evoked potentials. Note that VEPs elicited by either contralateral or ipsilateral eye stimulation resulted in lower amplitude values in the ethanol group (contra: 127.82 mV ± 18.46; ipsi: 83.31 mV ± 7.09) than the saline group (contra: 197.16 mV ± 19.5; ipsi: 120.17 mV ± 8.61). *p < 0.05; **p < 0.01.

At P25, contralateral bias was assessed by peak to trough measures of VEPs resulting from stimulation of each eye individually. Saline-treated animals exhibited the expected contralateral eye dominance with average contralateral bias indexes (CBIs, ratio of contralateral/ipsilateral response amplitude of each animal) of 1.6 ± 0.1 (*n* = 12). In this group, the average amplitude of contralateral and ipsilateral eye responses were 197.2 and 127.8 mV ± 18.5, respectively. Animals exposed to early alcohol exposure demonstrated an average CBI value of 1.5 ± 0.1 (Figure 1A) similar to saline controls. Yet, early ethanol exposure affected the strength of VEPs as alcohol-treated animals showed significantly lower amplitudes than controls in response to either contralateral (120.2 mV ± 8.6; *t* = 3.6, *p* < 0.01, df = 19) or ipsilateral (83.3 mV ± 7.1; *t* = 2.25; *p* < 0.05; df = 18) eye stimulation (Figure 1B).

### Visual acuity

**Figure 2:**
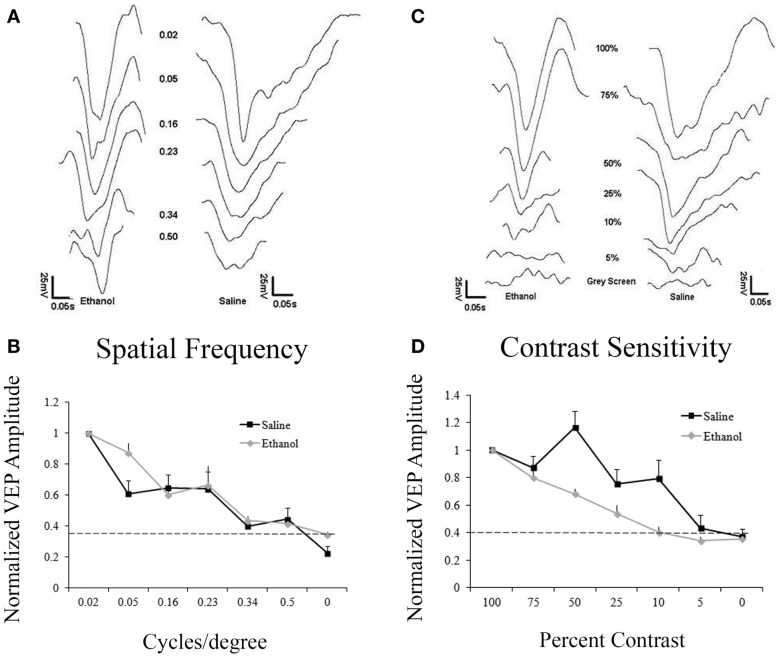
**Responses to varying contrast and spatial frequency**. **(A)** Representative VEP traces from an ethanol and a saline-treated animal after stimulation with different spatial frequencies (cycles/degree, cpd). **(A,B)** Saline and ethanol-treated animals showed similar decay in amplitudes after decreasing spatial frequencies were presented. Minimal responses at 0.5 cpd. Dotted line represents noise. **(C)** Representative VEPs from an ethanol and a saline-treated animal after visual stimulation at different contrasts. **(C,D)** As contrast decreases, saline-treated animals have a slow decrease in responses, showing a small response to 5% contrast, while ethanol-treated animals sharply drop-off their responses after 100% contrast, with no response occurring at 10% contrast.

Figure 2A shows responses to gratings from 0.02 to 0.50 cpd. Responses were normalized to the amplitude of the response to 0.02 cpd. Saline and ethanol-treated animals demonstrated similar spatial frequency acuity curves with maximal responses at 0.02 and 0.05 cpd, which decreased until responses could not be detected above noise at 0.50 cpd. In fact, a repeated measures ANOVA showed no differences between-groups for saline and ethanol-exposed animals (*F* = 0.6, df = 1, *p* = 0.4), but there was a significant linear effect within subjects (*F* = 331.2, df = 1, *p* < 0.001), indicating differences in response amplitude compared to changes in the cpd for each stimulation (Figure 2B). These results are compatible with the spatial frequency acuity responses described for mice (27).

Despite no change in spatial frequency acuity, we decided to explore contrast sensitivity in saline and ethanol-exposed animals. Animals were presented with 0.05 cpd stimuli with different levels of contrast from 100 to 5%. Contrast sensitivity responses were normalized to the amplitude of the response to 100% contrast. Control animals exhibited contrast sensitivity with peak responses occurring at 100 and 75% contrast. Response amplitudes then slowly decreased until there was barely any response above noise at 5% contrast. In contrast, ethanol-treated animals exhibited a precipitous drop-off of responses after 75% contrast, with no response detectable above noise at 10% contrast (Figure 2C). When this difference in contrast sensitivity was compared using a repeated measures ANOVA, there was a significant linear effect within subjects (*F* = 428.7, df = 1, *p* < 0.001), indicating differences in response amplitude compared to changes in contrast. Moreover, in between-groups measures, ethanol-exposed animals were shown to be significantly different from their control counterparts (*F* = 7.7, df = 1, *p* < 0.05) (Figure 2D).

### Electroretinography

**Figure 3:**
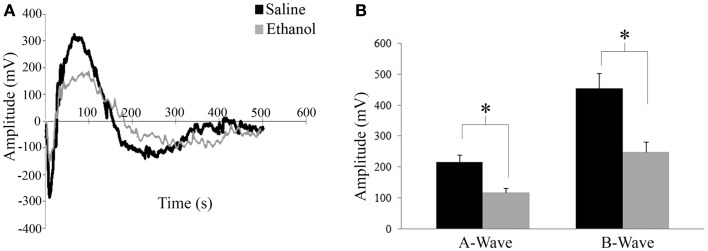
**Electroretinography**. **(A)** Retinal responses of saline and ethanol-treated animals. First a fast hyperpolarizing a-wave is seen, which represents the response of rod and cone photoreceptors to the light flash. This is followed by the positive b-wave, which represents depolarizing bipolar cell currents. **(B)** ERG recordings after dark adaptation show a difference in amplitude for both a-wave and b-wave responses between ethanol and saline groups. A-wave amplitudes (ethanol: 117 ± 14.5 mV versus saline: 216 ± 22.5 mV; *t* = 2.1; *p* < 0.01). B-wave amplitudes (ethanol: 249 ± 33.4 mV; saline: 454 ± 49.2 mV; *t* = 2.1, *p* < 0.01).

ERG was assessed in 12 saline and 6 ethanol-exposed animals. In order to assess the effect of early alcohol exposure on the visual system we first examined the retinal responses of ethanol and saline control animals at P30. Using a dark adapted ERG we were able to record the response of retinal cells to a flash of light. This response can be divided into two waves, as shown in Figure 3A. First, a fast downward deflection called a-wave is seen, which represents the hyperpolarizing responses of rod and cone photoreceptors to the light flash. The a-wave is followed by a slower upward deflection called b-wave, which represents the light-induced depolarization of ON-bipolar cells (28). A change in these currents could indicate a problem in phototransduction or synaptic transmission between photoreceptors and bipolar cells. Animals were dark adapted overnight, and ERGs were recorded in both eyes. A *t*-test showed that there was a difference in amplitude for both a-wave and b-wave responses between ethanol and saline groups. Animals treated with ethanol displayed a significantly decreased a-wave amplitude (117 ± 14.5 mV) compared to their saline-treated littermates (216 ± 22.5 mV; *t* = 2.1; *p* < 0.01). This decrease in response was also seen in b-wave amplitudes with ethanol-treated responses again being significantly smaller than controls (Ethanol = 249 ± 33.4 mV; Saline = 454 ± 49.2 mV; *t* = 2.1, *p* < 0.01) (Figure 3B). We found no significant difference between the a-wave/b-wave mean ratio of ethanol-treated animals (0.48 ± 0.01) compared to those treated with saline (0.48 ± 0.01; *t* = 0.3, df = 16, *p* = 0.7). This finding supports a more direct effect of alcohol on photoreceptors rather than an alteration of the synaptic transmission between photoreceptors and bipolar cells.

### Optical imaging of intrinsic signals

**Figure 4:**
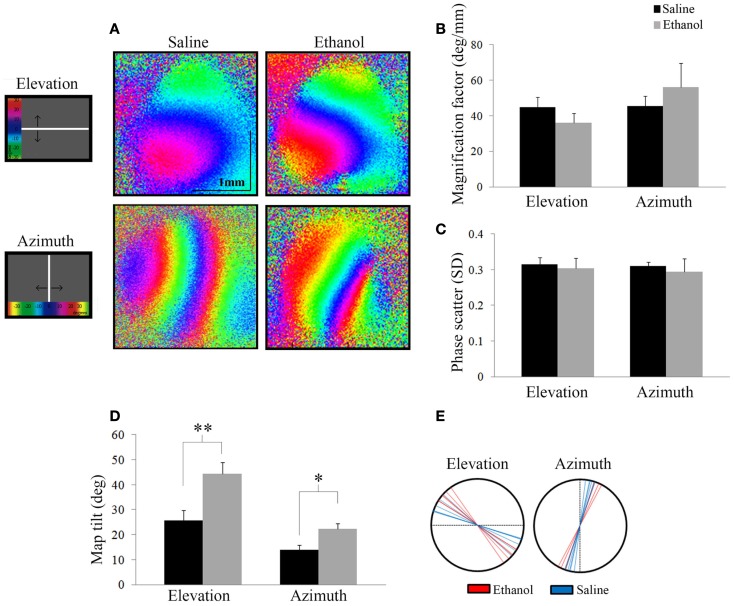
**Optical imaging of intrinsic signals**. **(A)** Representative elevation and azimuth retinotopic maps from different saline and ethanol-treated animals. To the left of the maps is a schematic of the corresponding visual stimuli presented, with a color scale representing degrees of the visual field. **(B)** Magnification factors of ethanol-treated animals were no different from that of saline-treated animals for elevation (*t* = 1.13, df = 12, *p* = 0.28) and azimuth (*t* = −0.86, df = 9, *p* = 0.4), signifying that the representation of the visual field in the cortex is similar between the two groups. **(C)** Phase scatter of cortical retinotopy shows no significant difference between saline and ethanol-treated animals in either elevation (*t* = 0.1, df = 12, *p* = 0.9) or azimuth (*t* = 0.3, df = 9, *p* = 0.8) maps. **(D)** The mean angles of map tilt were significantly different between ethanol and saline-treated animals, for both elevation (ethanol: 44.4 ± 4.5°, *n* = 7 versus saline: 25.7 ± 4.2°, *n* = 6; *t* = −3.02, df = 11, **p = 0.01) and azimuth maps (ethanol: 22.3 ± 2.3°, *n* = 4 versus saline: 14.0 ± 1.8°, *n* = 6; *t* = −2.85, df = 8, *p < 0.05). **(E)** Each line represents individual values (degrees) of map tilt in ethanol and saline-treated animals for both elevation and azimuth maps.

We investigated the effect of alcohol on the functional retinotopic organization of the primary visual cortex using an optical imaging technique of intrinsic signal mapping. A drifting white bar moving vertically or horizontally was used as the visual stimulus to obtain elevation or azimuth retinotopic maps, respectively. Figure 4A shows representative maps of ethanol and saline-treated animals. Three measurements were used to examine retinotopy – magnification factor, phase scatter, and map tilt.

Magnification factor (degree per millimeter) is defined as the degrees of space in the visual field represented per unit distance in the visual cortex. A lower magnification factor would mean a bigger representation of the visual field in an area of the visual cortex. For elevation, mice treated with ethanol did not show significantly different magnification factors compared to saline (Saline: 44.8 ± 2.2°/mm; *n* = 7 versus Ethanol: 36.0 ± 2.0°/mm; *n* = 7; *t* = 1.13, df = 12, *p* = 0.28). Similarly for azimuth, we found no significant change in magnification factors of ethanol-treated animals compared to saline (Saline: 45.6 ± 2.1°/mm; *n* = 7 versus Ethanol: 56.2 ± 6.7°/mm; *n* = 4; *t* = −0.86, df = 9, *p* = 0.4) (Figure 4B).

Scattering measures how well-defined phase bands are and high scatter is indicative of a poor quality map. Each phase in a retinotopic map (assigned to different colors) represents the region of the visual cortex that responds to the drifting white stimulus bar when it is in a particular location in the animal’s visual field. Note that the maps presented show clear separation between phases in both elevation and azimuth maps (Figure 4A). Therefore, lower phase scatter values would correspond to smooth progression across phases and a well-organized cortical retinotopy. To calculate phase scatter, we measured the difference (in units of standard deviation) between the phase value of individual pixels within the responsive visual cortex and the mean phase value of their neighboring pixels. There was no significant difference between the phase scatter in the elevation maps of ethanol-exposed animals (0.30 ± 0.03; *n* = 7) compared to saline (0.32 ± 0.02; *n* = 7; *t* = 0.1, df = 12, *p* = 0.9), nor in the azimuth maps of animals exposed to ethanol (0.29 ± 0.04; *n* = 4) compared to control (0.31 ± 0.01; *n* = 7; *t* = 0.3, df = 9, *p* = 0.8) (Figure 4C).

Finally, we investigated a possible effect of ethanol exposure on the orientation of primary visual cortex (V1) retinotopy by measuring the angle of the 0° phase band with respect to the X or Y axis, for elevation and azimuth maps, respectively. We found that this “map tilt” in elevation maps was almost twice that of control, with seven ethanol-exposed mice displaying a mean angle of 44.4 ± 4.5° compared to six saline animals that had a mean angle of 25.7 ± 4.2°(*t* = −3.02, df = 11, *p* = 0.01). Similarly, the azimuth maps of ethanol-exposed mice were significantly rotated compared to saline (Ethanol: 22.3 ± 2.3°, *n* = 4 versus Saline: 14.0 ± 1.8°, *n* = 6; *t* = −2.85, df = 8, *p* < 0.05) (Figures 4D,E).

## Discussion

Our findings demonstrate altered visual properties in adolescent animals previously exposed to ethanol during the third trimester equivalent of human gestation. We first demonstrated a decrease in VEP amplitudes in alcohol-treated animals when compared to controls, which might be a consequence of weak responses of individual neurons or due to a reduced number of cells. The latter seems to be more likely due to the dramatic effect of alcohol exposure in triggering neuroapoptosis, especially when the exposure is during the first 2 weeks after birth (22). Moreover, previous studies from our lab showed that early alcohol exposure does not change visual responsiveness of individual neurons in the primary visual cortex of the ferret (29, 30). Another possible cause of suppressed VEP amplitude could be a loss of feed-forward visual drive, due to a decrease in the myelinated fraction of axons within the optic nerve of animals exposed to a high dose of ethanol during the third trimester equivalent (31). This type of alcohol exposure also results in a decrease in cell number in the retinal ganglion layer of the retina and the dorsolateral geniculate nucleus (21, 32, 33). Additionally, the remaining retinal ganglion cells (RGCs) demonstrate reduced soma size, and dendritic length (32). The changes in RGC number and their properties may also contribute to the decrease in contrast sensitivity, as seen in VEPs, and the decrease in retinal response, as seen in ERG. Indeed, animals previously exposed to ethanol have been shown to have dose-dependent changes in rhodopsin, resulting in less visual input to the retina (16). Despite these changes, there did not appear to be an effect of early alcohol exposure on spatial frequency acuity.

In late gestation models of FASD, there are no gross ocular malformations as seen in first trimester equivalent ethanol exposure in animals and humans (34). In contrast, a single dose of ethanol on embryonic day 7 results in gross ocular malformations including abnormal lens development, defects of the cornea, and abnormal formation of the anterior chamber (6). Additionally, ethanol exposure throughout the first and second trimester results in a disruption of bipolar and horizontal cells of the retina, resulting in irreversible delays in retina development (35). These early retinal effects are responsible for many of the visual deficits seen in children with FASD, such as changes in acuity due to refractive error, and over-all poor visual function (1, 36). Interestingly, although our animals exhibit no gross eye malformations, we demonstrated an alteration in the receptive fields of V1.

Some of the most important developmental benchmarks of the visual system in rodents occur during the third trimester equivalent of human gestation. It is during this time that the retina fires Stage I, II, and III retinal waves, which are responsible for establishing the organization, binocularity, and connectivity of the structures within the visual system (37–39). The establishment of receptive fields in both V1 and the superior colliculus (SC) are strongly influenced by these retinal waves, as they are propagated simultaneously through the visual system (40). During our ethanol exposure paradigm, Stage III retinal waves are occurring. These waves are predominantly mediated by nicotinic acetylcholine receptors (nAChR), and have been shown to be required (along with correlated RGC activity) for the refinement of retinotopic maps (17, 37). Not surprisingly, ethanol modulates nAChR currents (41). This modulation of retinal wave currents could be responsible for our observed changes in V1 retinotopy.

In addition to retinal waves, ephrin gradients also help to establish retinotopic maps in both the SC and V1 (42). Early alcohol exposure between P4 and P7 has been shown to disrupt ephrin signaling pathways, which support the observed disruption in V1 retinotopic maps (43). As V1 and the SC share mechanisms underlying the establishment of their respective retinotopic maps (i.e., ephrin gradients, retinal waves), it would be interesting to test whether our model of alcohol exposure can also disrupt the retinotopy in the SC. Interestingly, the SC plays a major role in saccades, a visual process that is strongly affected in children with FASD (9, 11).

In conclusion, our findings show in a mouse model that alcohol exposure, restricted to the equivalent of the last months of human gestation, leads to clear visual processing deficits. These findings suggest that tests to assess visual function may be able to contribute to the diagnosis of prenatal alcohol exposure, especially when major craniofacial dysmorphologies are not present. Moreover, our results support the notion that FASD subjects can present visual problems even in absence of spatial frequency acuity deficits. It is conceivable that reduced contrast sensitivity, together with other visual problems, may aggravate learning problems observed in FASD children at school age.

## Conflict of Interest Statement

The authors declare that the research was conducted in the absence of any commercial or financial relationships that could be construed as a potential conflict of interest.
